# Dogs (*Canis familiaris*) stick to what they have learned rather than conform to their conspecifics’ behavior

**DOI:** 10.1371/journal.pone.0194808

**Published:** 2018-03-23

**Authors:** Markus Germar, Amira Sultan, Juliane Kaminski, Andreas Mojzisch

**Affiliations:** 1 Institute of Psychology, University of Hildesheim, Hildesheim, Niedersachsen, Germany; 2 Department of Psychology, University of Portsmouth, Portsmouth, Hampshire, United Kingdom; University of Exeter, UNITED KINGDOM

## Abstract

In recent years, an increasing number of studies has investigated majority influence in nonhuman animals. However, due to both terminological and methodological issues, evidence for conformity in nonhuman animals is scarce and controversial. Preliminary evidence suggests that wild birds, wild monkeys, and fish show conformity, that is, forgoing personal information in order to copy the majority. By contrast, chimpanzees seem to lack this tendency. The present study is the first to examine whether dogs (*Canis familiaris)* show conformity. Specifically, we tested whether dogs conform to a majority of conspecifics rather than stick to what they have previously learned. After dogs had acquired a behavioral preference via training (i.e., shaping), they were confronted with counter-preferential behavior of either no, one or three conspecifics. Traditional frequentist analyses show that the dogs’ behavior did not differ significantly between the three conditions. Complementary Bayesian analyses suggest that our data provide moderate evidence for the null hypothesis. In conclusion, our results suggest that dogs stick to what they have learned rather than conform to the counter-preferential behavior of others. We discuss the possible statistical and methodological limitations of this finding. Furthermore, we take a functional perspective on conformity and discuss under which circumstances dogs might show conformity after all.

## Introduction

In the 1950s, the seminal studies of social psychologist Solomon Asch [1] showed that humans tend to adopt a unanimous but clearly erroneous majority opinion in a considerable number of cases, thereby suggesting that humans tend to forgo personal information by conforming to a majority (cf. [2]). This classic “Asch-effect” was replicated across different cultures and age levels [3,4] and provoked a larger number of further investigations on majority influence in humans [5].

In biology, the term conformity was mainly informed by cultural evolution theory and social learning research [6]. Here, conformity was defined as an individuals’ tendency to disproportionately adopt the majority choice, when there is no individual preference (i.e., the individual weights all options equally; e.g., when an individual is totally naïve). Cultural evolutionary modeling showed that conformity can be adaptive by exploiting the “wisdom of the crowd”, and that conformity is an important driving force behind cultural diversification since it promotes within-group homogeneity and between-group heterogeneity [2,7]. Research under this framework suggested that conformity is better understood as a context dependent adaptive strategy rather than a purely “irrational” herding behavior (see [2,6,8,9] for reviews). In line with this idea a recent study showed that humans’ tendency to conform increased adaptively with the number of majority members, consensus among majority members, confidence of subjects, task difficulty, and performance of majority members, providing evidence for the notion that conformity is regulated by adaptive social learning strategies [10].

In recent years, more and more studies have investigated majority influence in nonhuman animals (e.g., [11–15]). As discussed in current reviews of the field [2,6,8,9], due to large differences in definitions of conformity as well as due to methodological issues, evidence for conformity in nonhuman animals is scarce and controversial (for a brief review of these studies, see below). In the present study, we aimed to examine whether dogs (*Canis familiaris)* show conformity. To the best of our knowledge, this has not been tested before, although dogs have become a popular model for animal social cognition and behavior [16,17]. In what follows, we first explain the conceptual framework we used to define conformity. Second, we briefly summarize studies that have recently investigated conformity in other nonhuman animals and have thereby informed our experimental design and methodological procedure. Third, we briefly present results from dog research, and explain the basic rationale of our study.

### Conceptual background

To date, the interdisciplinary perspective on conformity and majority influence has suffered from the fact that different disciplines have used different definitions and, consequently, have investigated different types of behavior [2,6,8]. For example, social psychologists typically use the term conformity to describe behavior that is analogue to the Asch-effect (i.e., to forgo personal information in order to go along with the majority). In contrast, biologists tend to define conformity as the disproportionate tendency to copy the behavior that is most frequent in a given population ([7], cf. [2]).

To overcome this problem, van Leeuwen and Haun [2,9] proposed a conceptual framework, where majority influence refers to “all instances where the very presence of a majority affects the behavior of observers. Importantly, here, we define these effects in terms of behavioral end results, not mechanisms” [2]. More specifically, van Leeuwen and Haun [2] define two types of end results. Conformist transmission refers to “the disproportionate tendency of naïve individuals to copy the behavior of the majority” ([2], italics added). In contrast, conformity refers to “the tendency to forgo personal information by adopting the cultural variant that is used by the majority” ([2], italics added). Both end results should be distinguished from the possible underlying causal mechanisms. Thus, both can be caused by either the observed majority behavior per se (i.e., the copy-the-majority heuristic), by characteristics that are typically confounded with the majority, or even social learning without any specific bias [18,19]. One example is that the majority behavior is mostly the most frequent behavior, so that random copying can give rise to conformity. Another example is that a majority is often comprised of the most skillful or prototypical members of a population; hence, heuristics tuned toward such characteristics could also lead to conformity. Finally, according to van Leeuwen and Haun [9], it is necessary to show that individuals of a species have a higher tendency to forgo personal information when they are confronted with a majority than with a single conspecific (i.e., that they show a specific susceptibility to majority influence rather than a general susceptibility to social influence) in order to establish conformity in a species.

In the present study, we investigated conformity in dogs, adopting the conceptual framework by van Leeuwen and Haun [2]. Hence, we tested whether dogs would forgo personal information by adopting the majority behavior. More specifically, we used an experimental paradigm that allowed us to test whether dogs show a greater tendency to forgo their personal information when confronted with the behavior of three conspecifics (i.e., a majority) rather than a single conspecific. Thereby, we were able to examine whether dogs show conformity rather than a general susceptibility to social influence [9].

### Empirical background

In nonhuman primates, the first evidence for majority influence came from studies using the “reversion design” (e.g., [6], cf. [2]). These studies showed that individuals who had invented a new behavioral strategy reverted back to the majority strategy, which was interpreted as evidence for conformity. However, this interpretation was criticized because of several limitations. One important limitation was that the majority strategy was initially acquired through social learning from a single individual in the population. Thus, reverting back to this strategy could simply be due to individual behavioral conservatism rather than due to the majority behavior (see [2] for an detailed discussion of all limitations). Two recent experimental studies [11,20], which did not suffer from these limitations and which followed the conceptual framework described above, revealed that chimpanzees do not show conformity. In contrast, a recent field study [21] revealed that male wild vervet monkeys (*Chlorocebus aethiops*), who had learned a certain foraging preference in one group and subsequently immigrated to another group, showed conformity by abandoning their previous preference and switching to the preference represented by the local majority.

Galef and Whisken [14] showed that rats (*Rattus norvegicus*) increased their consumption of a certain diet after they were in contact with one (Experiment 2) or two conspecifics (Experiment 1) who had recently eaten this diet. Importantly, rats had learned to avoid this diet or to prefer another diet, respectively, before they had contact with their conspecifics. In other words, rats forwent their personal preference and adopted the preference of their conspecifics. Interestingly, this effect was already observed when rats had contact with only one demonstrator. Thus, the results suggest that rats are generally susceptible to social influence but it remains unclear whether they show conformity (i.e., are specifically susceptible to majority influence, cf. [9], see conceptual background).

In a study by Pike and colleagues [15], sticklebacks first learned to prefer one of two foraging patches because it offered more food than the alternative. Then, these observers were confronted with demonstrators who shoaled at both patches. The number of demonstrators at each patch was manipulated. The results revealed that observers disproportionally adopted the foraging patch where the majority of demonstrators had shoaled even when this patch was not the one preferred by the observer. Hence, sticklebacks show conformity by forgoing their personal preference and adopting the majority preference.

A recent field study on great tits (*Parus major*, [13]) showed that individuals acquired an experimentally introduced innovation by disproportionately copying the majority. Furthermore, individuals who had learned a variant in one population and subsequently immigrated to another population quickly switched to the variant represented by the local majority. Hence, great tits seem to show conformist transmission as well as conformity. However, it is unclear whether individuals actually observed the actions of the demonstrators or conspecifics, respectively. Thus, the way in which the behavioral innovation was transmitted from one individual to the other was not uncovered.

Taken together, so far only a few studies have investigated and provided evidence for conformity in different nonhuman species (i.e., great apes, monkeys, sticklebacks, and great tits; for a more detailed review, see [9]). Thus, conformity evolved independently and is probably generated by different mechanisms in different species. However, due to the thin empirical basis, more studies from other species are needed to complement the comparative perspective. This is why we decided to investigate whether dogs (*Canis familiaris*) show conformity in the present study.

### Present study

It is typically assumed that due to selection processes during domestication, dogs became more socially oriented and relatively agreeable [22]. In particular, they have been found to be sensitive to and can profit from their conspecifics’ behavior [16], although there is currently a debate about whether dogs can show complex forms of social learning (e.g., [23]). Furthermore, dogs have been found to show prosocial tendencies towards conspecifics [24]. However, as dogs’ evolution was strongly shaped by domestication, it is also possible that they developed a more anthropocentric orientation, which does not include a specific sensitivity for the behavior of their conspecifics’ majority. Hence, it remains to be examined whether dogs forgo personal information to adopt the majority behavior, thereby showing conformity.

To address this question, we took a similar approach as the studies described above [11–15]. First, dogs learned to prefer one simple behavioral response over another (i.e., bypassing a wall on the right instead of on the left side). As in the previous studies, this learning experience constituted the personal information that was inconsistent with the information provided by the majority presented later. Second, we confronted them with counter-preferential behavioral responses of either no (control condition), one, or three conspecifics (experimental conditions; e.g., one or three conspecifics bypassed the wall on the left side). If dogs chose to bypass the wall in the non-preferred direction more often in the experimental conditions than in the control condition, this would indicate unspecific counter-preferential social influence. More importantly, if dogs switched their responses more often after having observed three conspecifics rather than one, this would indicate conformity (i.e., specific susceptibility to majority influence, cf. [9]). It is worth noting that this effect could not be attributed to the observed frequency of the demonstrated response, as it was the same in both condition (i.e., three demonstrations, cf. [12] see Methods).

Furthermore, we chose the task described above because it is structurally similar to the widely used detour paradigm, where dogs have to move around a (V-shaped) fence in order to get a reward [23]. Several studies have shown that dogs are more successful in this task (i.e., bypassing the fence more often), after watching demonstrations from conspecifics, humans or even moving objects, respectively; which is most probably due to social learning via stimulus enhancement [23]. Thus, we assume that dogs in our task also have the capacity to use the responses of conspecifics to inform their behavior. In contrast to the detour paradigm, the dogs in our study were not naïve to the task when they observed the conspecifics because we were not interested in whether dogs could merely bypass the wall, but whether dogs deviated from the direction they had learned to bypass the wall previously.

## Methods

### Sample

Since there are no past studies on conformity in dogs, we did not determine our sample size prior to data collection. We rather aimed to test as many dogs as possible during the time of data collection (i.e., four months). Notably, our final sample size exceeded those normally used in experimental studies on social learning in dogs (e.g., [23,25]). Dogs and their owners were recruited via personal contact, Facebook and newspaper articles. Selection criteria to participate were that dogs had to be older than one year, without any medical conditions, and without any acute psychological or behavioral problems (e.g., chronic aggressive behavior, high levels of anxiety or avoidance behavior). Additionally, an experimental session was stopped when a dog showed the latter problems during participation. One-hundred-and-seventeen dogs (*M*_age_ = 5.02 years, *SD* = 2.96, 62 females) of different breeds (32 mixed breed, for details see: https://osf.io/dgtxt/) participated as observers in our study. Seventeen dogs were trained to serve as demonstrators (*M*_age_ = 5.74 years, *SD* = 4.05, 12 females) in the experimental task. All dog owners provided their verbal consent to the study at the beginning of the experimental session. The approval of an institutional review board was not necessary prior to data collection because no special permission for the use of animals in purely behavioral or observational studies is required in Germany (TierSchGes §7 and §8). Although our study was an experiment, our experimental manipulation (i.e., confronting dogs with the behavior of no, one or three demonstrators, respectively) was not invasive according to the guidelines by the Association for the Study of Animal Behaviour (ASAB). During an experimental session, dogs were confronted with a new unknown situation, unknown dogs (i.e., the observers), and unknown humans (i.e., the experimenters). However, their owners were always present and dogs were carefully familiarized with the observers and the experimenters (see Methods). No dog was food deprived at any moment: Dogs were fed according to their owners’ daily routine. Dogs received regular breaks and the opportunity to drink water. Observations were terminated if dogs showed any signs of stress or anxiety. After data collection, an independent animal protection officer confirmed that our study has met all ethical standards.

### Experimental setting and design

**Fig 1:**
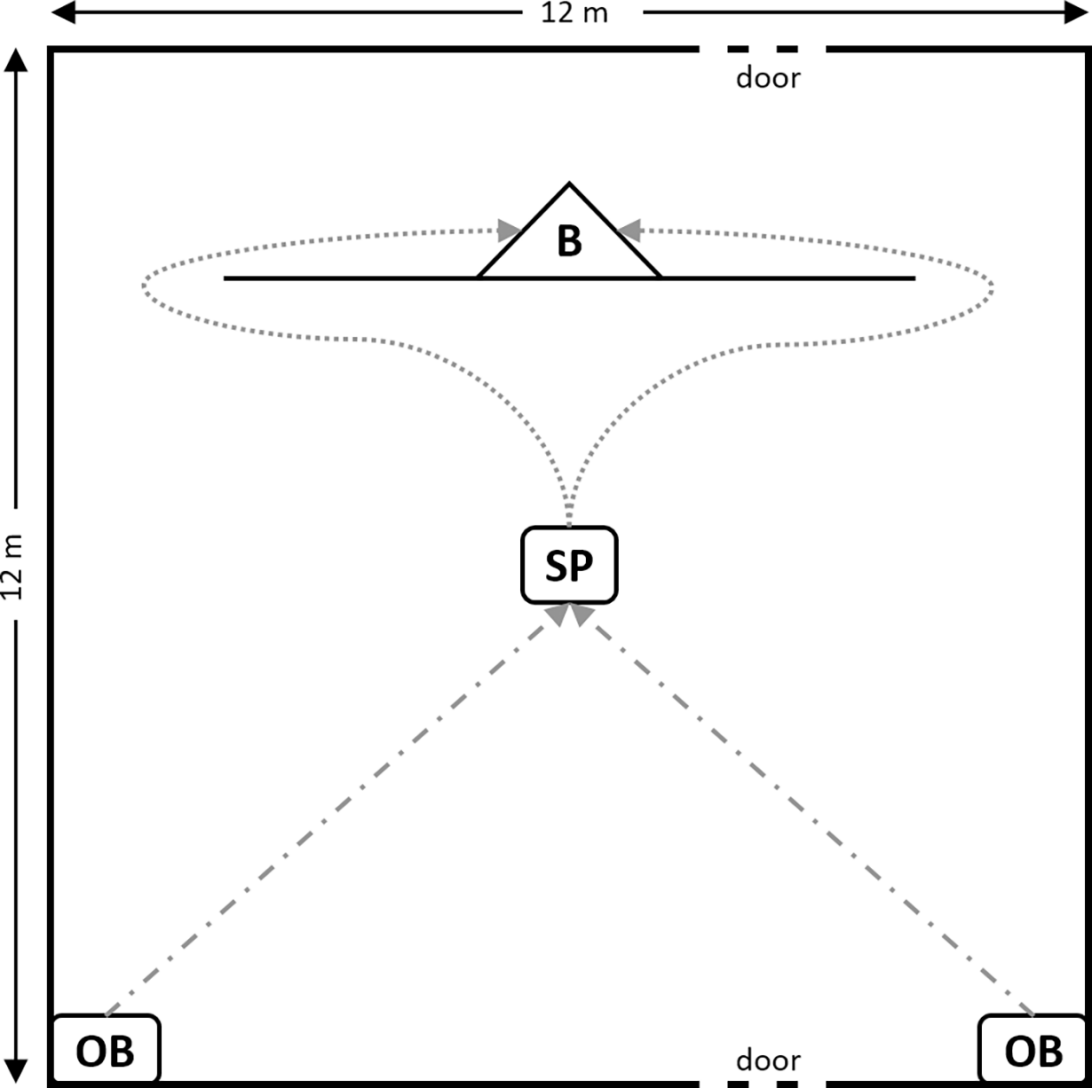
Experimental set-up. OB = possible observation position, B = box behind the wall, in which Experimenter 1 stood during trials, in order to not be visible to the observer, SP = starting position, dashed lines = possible direction an observer was led to the starting position, dotted lines = possible options for an observer to bypass the wall.

The experiment took place inside a small riding hall (see Fig 1 for graphical representation of the experimental set-up), where a 9 m (width) × 2 m (height) wall was installed 2 m from the rear of the hall. In preparation for the experiment, the demonstrators were individually trained to bypass the wall either on the left or on the right side of the wall (eight right/nine left).

At the beginning, observers were trained to prefer bypassing the wall either on the left or the right side (i.e., preferred direction; for details of the training, see below). Then, during three experimental trials, each observer saw either no (control condition), one or three demonstrators (experimental conditions) bypassing the wall in the direction opposite to their preferred direction. In line with Haun et al. [12], only the number of demonstrators differed between the experimental conditions while keeping the number of demonstrated responses constant. Hence, observers saw either one demonstrator showing his response three times on each trial or three demonstrators showing their respective response once on each trial.

Demonstrators entered the hall through a door near the center of the front of the hall. To prevent direct contact between demonstrators and the observer, observers watched demonstrators not from the center but either from the front left or front right corner of the hall (i.e., observation position, Fig 1). After that, observers were led to the starting position at the center of the hall and unleashed. When the observer bypassed the wall in the preferred versus opposite direction, this was coded as a *stay* versus a *switch* response, respectively. Depending on the preferred direction and the observation position, each observer was either directed towards a stay or a switch response when he was led to the starting position. To account for possible effects of this initial direction on observers’ responses, we collapsed preferred direction and observation position to the factor start direction (towards a stay vs. a switch response).

In sum, observers were randomly assigned to complete each of the three task trials in one of three experimental conditions (no, one, or three demonstrators) and from one of two start directions (towards stay vs. switch response). Thus, we employed a 3 (condition: no vs. one vs. three demonstrators) × 2 (start direction: towards stay vs. switch response) design with the first two factors manipulated between-subjects and the last factor manipulated within-subjects.

### Procedure

#### Training

The experiment was conducted in individual sessions for each observer. At the beginning of each experimental session, the observer was familiarized with the riding hall and the two female experimenters.

Then, Experimenter 1 built up the observer’s preference. First, Experimenter 1 presented the food reward (Platinum® Chicken) ten times along with a verbal amplifier (i.e., the word “bing”) outside the hall. Thereby, the observer learned to associate the amplifier with the food reward. The verbal amplifier was used to enable Experimenter 1 to immediately reward the observer’s behavior during the training in the hall.

After that, Experimenter 1, 2, and the observer entered the hall; Experimenter 1 trained the observer to bypass the wall in the desired direction using the shaping technique. Shaping was used to guarantee that the observers autonomously learned their preference in small steps (e.g., [26]). Experimenter 1 supported the observers’ learning process only by three means: (a) demonstration (i.e., going to and then standing at the side of the wall, at which the observer was supposed to bypass the wall), (b) using the verbal amplifier and/or (c) the food reward. Importantly, there was neither direct communication between Experimenter 1 and observer (e.g., Experimenter 1 did not point to the desired direction, did not directly speak to, and did not look at the observer) nor direct social contact (e.g., Experimenter 1 presented the food reward in a bowl and not from her hand). Additionally, the support was successively reduced. In the end, observers only received the food reward and had no visual contact with Experimenter 1.

The training trials were executed as follows: As in the experimental trials, Experimenter 2 first led the observer from his observation position to the starting point and unleashed him. Then, the observer’s behavior was rewarded depending on the stage of training. In the first stage, the observer could see Experimenter 1 going to and then standing at the desired side of the wall, before the trial was started. When the observer moved in this direction he or she was immediately rewarded with the verbal amplifier and then with the food reward. In the following trials, the observer was only rewarded in this way when he or she moved further in the correct direction than in the respective trial before. When the observer accomplished moving to the correct side of the wall the next training stage began. At the beginning of a trial, Experimenter 1 was hence not visible to the observer because she stood behind the wall. The observer was only rewarded for going around the wall on the correct side, where he or she could then see Experimenter 1. When the observer accomplished this, the next training stage began, where the trials were executed exactly as the experimental trials. At the beginning of a trial, Experimenter 1 stood in a box, which was part of the back side of the wall. Thus, Experimenter 1was not visible to the observer for the whole trial. Importantly, the observer could not anticipate that Experimenter 1 was behind the wall, because she entered the hall through a back door behind the wall and went into the box. The observer was rewarded only with the food reward but no longer with the verbal amplifier and only when he or she went around the wall on the correct side. Experimenter 1 could see whether the observer bypassed the wall correctly, in which case she administered the food reward in a bowl by sliding it under the screen of the box before the observer arrived. Next, it was tested whether the observer showed the desired response three times in a row (i.e., the training criterion). When this was not the case, the training was restarted at the preceding stage. Training was rated as successful when the observer reached the training criterion (*M*_*time*_ = 22.83 min, *SD* = 11.07). Since observers’ behavior was successively shaped as described, the observer had no opportunity to bypass the wall on the wrong side during training.

After the training trials, observers in the experimental conditions met the demonstrators in a controlled procedure. Guided by their respective owners, dogs approached each other slowly on a field outside the riding hall. This was done (a) to exclude that observers would follow demonstrators only out of curiosity and (b) to test whether dogs showed any sexual interest or aggressive behavior (in which case observers were excluded). This meeting in the experimental conditions took about 9 minutes (*M*_*time*_ = 8.97, *SD* = 4.07); in the control condition there was a 10 minute break instead.

#### Experiment

Thereafter, observers completed the experimental task, which was recorded with two video cameras. A trial started with the observer situated at his observation position (i.e., either at the front left or front right corner of the hall) and leashed by Experimenter 2. In the experimental conditions, a demonstrator entered the hall guided by his owner, was unleashed at the starting position, and showed a response opposite to the observer’s preferred direction established in the training phase. Then, the demonstrator was let out through the back door and rewarded by Experimenter 1, who was not visible to the observer due to the wall. Simultaneously, the demonstrator’s owner left the hall through the front door. Depending on the experimental condition, this line of action was repeated two additional times with the same demonstrator or two different demonstrators. Subsequently, Experimenter 1 entered the box, which was part of the wall. Then, the observer was led to the starting position and unleashed. If the observer bypassed the wall in his preferred versus opposite direction, this was coded as a stay versus a switch response, respectively. Stay but not switch responses were rewarded, because we wanted to use switch responses as an indicator of genuine conformity over all trials. If we had rewarded both types of responses or none of them, switch responses could have also been attributed to individual learning experience in past trials. Thus, if observers showed switch responses after being rewarded for a switch response in the trial before or after not being rewarded for a stay response in the trial before, respectively, it would be unclear whether this switch response was due to conformity or due to the individual learning experience in the trial before. After the observer’s response, Experimenter 2 re-leashed him, brought him back to the observation position and the next trial began. In the control condition, the procedure was virtually the same. However, instead of watching the demonstrators’ responses, the observer had to wait 90 seconds, which corresponded to the duration of the demonstrations in the experimental conditions.

During the experimental trials, we went to great lengths to minimize any form of human influence on the observers’ behavior (e.g., any non-verbal cues or communication). First, Experimenter 1 (the trainer) was not visible during the experimental task and administered the food reward from within the box, which was part of the wall. Second, the influence of Experimenter 2 was restricted to (un-)leashing the observers and leading them to the observation or starting position, respectively. Similarly, the influence of demonstrators’ owners was restricted to leading their dog to the starting position and unleashing him. Finally, the observers’ owners were seated in the corner of their dogs’ observation position. They were faced to the wall and read a newspaper, thereby blocking eye contact with the observer.

### Statistical analyses

Data preprocessing and preliminary analyses were conducted using R [27]. For our main analyses, we conducted traditional frequentist analyses (analyses of variance, ANOVAs and *χ*^*2*^-tests, see below) accompanied with their Bayesian counterparts with default priors using the open JASP software [28–31]. We decided to conduct Bayesian analyses for two reasons. First, as explained above, our sample size was not based on any statistical rationale but rather on practical consideration. In this case, Bayesian analyses are preferred because they allow researchers to analyze their data irrespective of an a priori rationale behind the sample size [29]. Second, and more importantly, Bayesian analyses allowed us to explicitly quantify the evidence for the alternative hypothesis (i.e., there is a difference between conditions, H1) or the null hypothesis (i.e., there is no difference between conditions, H0). Specifically, we report Bayes factors expressing the probability of the data given H1 relative to H0 (*BF*_10_, a value of 1 indicates that the data is equally likely under both hypotheses). Furthermore, we apply the descriptive classification scheme for the interpretation of *BF*_10_ values ranging as developed by Wagenmakers and colleagues [28]. *BF*_10_ values ranging from 1 to 3, from 3 to 10, from 10 to 30, from 30 to 100, and > 100 indicate anecdotal, moderate, strong, very strong, and extreme evidence for the H1, respectively. In contrast, BF_10_ values ranging from 1 to 3, from 1/10 to 1/3, from 1/30 to 1/10, from 1/100 to 1/30, and < 1/100 indicate anecdotal, moderate, strong, very strong, and extreme evidence for the H0, respectively. For example, a *BF*_10_ of 4 would indicate that the data is 4 times more likely under the H1 (i.e., moderate evidence for H1). In contrast, a *BF*_10_ of 0.25 would indicate that the data is 4 times more likely under the H0 (i.e., Bayes Factor for the null hypothesis, *BF*_01_ = 1/*BF*_10_ = 1/0.25 = 4). Note that the *BF* is constrained by the acquired sample size, so that *BF*s based on small samples cannot indicate strong evidence (see [28,29] for a more detailed introduction to Bayesian analyses). In contrast, in the frequentist framework, significant effect sizes based on small samples strongly overestimate the real effect size (cf. [32]). The data of this article are available at: https://osf.io/dgtxt/.

## Results

### Preliminary analyses

Forty-one dogs had to be excluded from all analyses because they did not complete the experiment: Twenty-five dogs showed stress or anxiety, 13 did not reach the training criterion, and three were excluded for other reasons (i.e., high outdoor temperature and/or no interest in reward). All exclusions were based on observations and decisions by Experimenter 1, the second author, who is a very experienced animal psychologist and dog trainer. Excluded and included dogs did not differ in sex, *χ*^*2*^ (1, *N* = 117) = 0.24, *p =* .63, or age, *t*(115) = 0.50, *p* = 0.62. Both groups were evenly distributed over conditions, *χ*^*2*^ (2, *N* = 117) = 0.34, *p =* .84. The final sample was *N* = 76 (*M*_age_ = 4.92, *SD* = 2.93, 39 females).

### Main analyses

To test whether dogs differed in their behavioral responses between conditions, we ran a 3 (condition: control vs. one demonstrator vs. three demonstrators) × 2 (start direction: towards stay vs. switch response)–ANOVA with the percentage of switch response across the three trials as the dependent variable (see Table 1 for descriptive statistics). There was no main effect for condition, *F*(2,68) = 0.69, *p* = .50, ƞ^2^_p_ = .02, indicating that dogs in the control condition showed as many switch responses (*M =* 20.83%, 95% CI [5.48, 36.19]) as dogs that observed one (*M =* 25.93%, 95% CI [14.42, 37.43]) or three demonstrators (*M =* 32.24%, 95% CI [19.81, 44.67]), respectively. However, there was a significant main effect for start direction, *F*(1,68) = 42.00, *p* < .001, ƞ^2^_p_ = .38, revealing that there were almost no switch responses and hence no behavioral variability when dogs’ start direction was oriented towards a stay response (*M =* 1.60%, 95% CI [-9.14, 12.32]), whereas behavioral variability was present when dogs’ start direction was oriented towards a switch response (*M =* 51.08%, 95% CI [40.26, 61.90]; interaction effect n.s., *F*(2,68) = 0.24, *p* = .79, ƞ^2^_p_ = .01). Without speculating about the causes of this floor effect in the former condition at this point (see Discussion), we next explored whether there were any differences between conditions by running a one-way-ANOVA using only the data from dogs in the latter condition (i.e., when dogs were directed towards a switch response). Again, there was no effect for condition, *F*(2,35) = 0.41, *p* = .66, ƞ^2^_p_ = .02.

**Table 1:** Descriptive statistics for the percentage of switch responses across trials.

start direction	condition	Mean	SD	N
towards stay	control	0.00	0.00	9
	one demonstrator	0.00	0.00	13
	three demonstrators	4.76	12.10	14
towards switch	control	41.67	42.73	8
	one demonstrator	51.85	41.57	18
	three demonstrators	59.72	46.85	12

To quantify how strong the lack of main effects for condition supported the null hypothesis, we ran complementary Bayesian ANOVAs. The Bayesian ANOVAs on the complete design gave us the *BF*_10_ for each of the four possible models (i.e., one for each main effect, one for both main effects, and the full model with both main effects and the interaction) against the null model (see Table 2). The best model (i.e., having the highest *BF*_10_) was the model only containing the main effect for start direction. Comparing this model with the remaining models (i.e., calculating *BF*_comparison_ = *BF*_10, start direction_ / *BF*_10, other model_) yielded that there was at least moderate evidence (*BFs*_comparison_ > 5.22) for this model over each of the other ones. Importantly, the model containing the main effect for condition was the worst model. Its *BF*_10_ of 0.165 actually indicated moderate evidence for the H0 (i.e., the data is six times more likely to be observed under the null model than under this main effect model). These results were supported by an analysis of effects, which revealed the *BF* for each effect across all models (i.e., *BF*_inclusion_, see Table 3). There was extreme evidence (*BF*_inclusion_ > 100) for including the factor start direction, whereas there was moderate evidence against including the factor condition (*BF*_inclusion_ = 0.157). Additionally, a Bayesian ANOVA using only dogs in the condition in which observers were directed towards a switch response revealed an *BF*_10_ of 0.249 for the model including the main effect for condition, thereby again indicating moderate evidence for the H0 (i.e., the data is four times more likely to be observed under the null model than under this model).

**Table 2:** Bayesian ANOVA, model comparison.

Models	P(M)	P(M|data)	BF _M_	BF _10_
Null model	0.200	1.367e -7	< .001	1.000
start direction	0.200	0.809	16.955	5920000.000
condition	0.200	2.250e -8	< .001	0.165
start direction + condition	0.200	0.155	0.733	1134000.000
start direction + condition + start direction ✻ condition	0.200	0.036	0.149	262883.822

**Table 3:** Bayesian analysis of effects.

Effects	P(incl)	P(incl|data)	BF _Inclusion_
start direction	0.600	1.000	4188000.000
condition	0.600	0.191	0.157
start direction ✻ condition	0.200	0.036	0.149

Since stay but not switch responses were rewarded, dogs differed in their reinforcement history in the second and the third trial depending on their responses in the preceding trials. Consequently, it can be argued that dogs differed in the preconditions under which they responded in these trials, and, hence, only responses in the first trial could be considered as being comparable between dogs. To overcome this issue, we ran *χ*^*2*^-tests and their Bayesian counterparts on a 3 (condition: control, one demonstrator, three demonstrator) × 2 (response: switch vs. stay)—contingency table (see Table 4). Similar to our ANOVAs, we ran one analysis for the complete sample (collapsing over the factor start direction) and one analysis only for dogs who were directed towards a switch response. In both samples, the *χ*^*2*^-test did not reach significance, *χ*^*2*^s < 1, *p*s > .61, indicating that conditions did not differ in the frequency of switch responses in the first trial. Thus, even when we excluded the influence of inter-individual differences in reinforcement history, there were no differences in the likelihood of switch responses between the three conditions. Furthermore, the Bayesian analyses revealed moderate evidence for the H0 in both samples. More specially, the *BF*_10_(independent multinomial) was 0.148 for the full and 0.252 for the sub-sample, indicating that the data were 6.76 and 3.97 times more likely to be observed under the H0 than under the H1, respectively.

**Table 4:** Contingency tables for the complete and each sub-sample.

		response in trial 1		
start direction	condition	stay	switch	Total
towards stay	control	8	0	8
	one demonstrator	12	0	12
	three demonstrators	12	2	14
	Total	32	2	34
towards switch	control	4	3	7
	one demonstrator	8	9	17
	three demonstrators	5	7	12
	Total	17	19	36
Total	control	12	3	15
	one demonstrator	20	9	29
	three demonstrators	17	9	26
	Total	49	21	70

In sum, our analyses revealed moderate evidence for the hypothesis that there were no differences between conditions (i.e., the H0). Thus, dogs did not show conformity when confronted with counter-preferential behavior of their conspecifics.

## Discussion

Dogs are highly social animals. However, so far it is unknown whether they show conformity, which refers to the tendency to forgo personal information (e.g., learning experience) by adopting the majority’s behavior [2]. Our study is the first to examine whether dogs conform to their conspecifics’ behavior rather than stick to what they have previously learned. Specifically, after dogs had acquired a behavioral preference, we confronted them with counter-preferential behavior of either no, one or three conspecifics. Our results revealed that dogs did not differ in the likelihood of switching their responses across the three conditions. Thus, we found no evidence for conformity in dogs in our study. More specifically, we found that dogs were neither generally susceptible to social influence nor specifically susceptible to majority influence (cf. [9]). Before we reflect on the implications of this finding, we will discuss the potential limitations of our study. We will begin with general factors that could have prevented us from finding conformity in dogs (i.e., the sample size, the effect of start direction). Following van Leeuwen and Haun [2], we will then discuss our operationalization of the two central aspects of conformity, which are (a) the personal information dogs had (i.e., the training and learning experience), and (b) the social information dogs were confronted with (i.e., the demonstrators and the method of demonstration).

### Sample size

We had to exclude 41 out of 117 dogs (i.e., 35%). Most exclusions were due to the fact that dogs showed signs of anxiety or stress, or because dogs did not reach the training criterion. Unfortunately, we cannot tell whether our exclusion rate is high because, as was pointed out in a recent review [33], past studies on dogs did not systematically report the exclusion rate and/or exclusion criteria. We attribute our exclusion rate to three intertwined factors. First, we sampled dogs from a wide range of socio-demographic characteristics and living conditions (e.g., past training experience, frequency of contact with unknown dogs and humans, function, …) without applying strict a priori participation criteria. Hence, it is conceivable that our training procedure was not effective for a subgroup of dogs. Second, dogs interacted with humans (i.e., the experimenters) during the experimental sessions who were largely unknown to them and who acted socially neutral towards them (e.g., did not address them directly) while their owners did not interact with them at all. Since dogs are very sensitive to humans [34], at least some dogs might have been alienated by the unnatural human behavior. Third, because we were aware of the latter factor, we applied a conservative exclusion strategy by excluding dogs as soon as they showed only minimal signs of stress or anxiety. In such cases, dogs were immediately brought outside the hall to their owner, which led to a fast recovery in all cases. In no case did excluded dogs need medical or psychological treatment.

Our final sample size (*N* = 76) fits well in the range of samples used in animal studies investigating conformity (e.g., [11,14,15,20,21]) and is larger than in 84% of studies on canine cognition, which had sample sizes of fewer than *N* = 50 [33]. Nevertheless, the statistical power of our study can be considered rather small for establishing the non-existence of conformity in dogs via traditional frequentist analyses. To illustrate this problem, we calculated the power of our study when assuming a significance level of .05 and a small (*f* = .1), medium (*f* = .25) or large effect (*f* = .4), respectively. For the complete sample (N = 74), the corresponding power was .11, .45, or .86, respectively. For the sub-sample (N = 36), where dogs were directed towards a switch response, the corresponding power was .08, .23, or .52, respectively.

Our Bayesian analyses did not suffer from this problem and allowed us to quantify the evidence for the non-existence of conformity in dogs. The results referring to differences between the three conditions show that our data was roughly four to six times more likely to be observed under the H0 than under H1, which can be considered as moderate evidence for the H0 [28].

Nonetheless, both analytical approaches suggest that more studies with larger sample sizes are needed to increase our cumulative knowledge about conformity in dogs.

### Effect of start direction

Our results showed that dogs tended to respond congruently to their start direction. This was possibly due to the start direction focusing the dogs’ attention towards the side of the wall in the corresponding line of sight, which could have influenced dogs’ responses in a similar way as stimulus enhancement [23]. This led to a floor effect (i.e., almost no switch responses) when dogs were directed towards a stay response. However, when the start direction could have facilitated switch responses (i.e., when dogs were directed towards a switch response) there was no comparable ceiling effect, which could have suppressed differences between the control and experimental conditions. Additional analyses revealed that there was no evidence for conformity even when including only dogs that were directed towards a switch response. Thus, the effect of start direction cannot explain why we did not find any evidence for conformity.

### Conformity and personal information

Since conformity implies forgoing personal information, it is important to consider what constitutes this personal information and how strong it is [2,9].

Following previous studies on conformity in nonhuman animals [11,14,15], we operationalized personal information as an individually learned preference. However, in contrast to these studies, dogs learned a behavioral response through a stepwise training procedure (i.e., shaping). This training was executed by Experimenter 1 and thus involved human-dog interaction, albeit we sought to keep this interaction to a minimum. It is possible that these training characteristics (i.e., stepwise training, human interaction) led to a particularly strong preference in dogs, which could not be overwritten by social information (i.e., the demonstrators’ behavior). Additionally, the social information probably had a rather small intensity compared to the training. Interestingly, Galef and Whiskin [14] showed that an experimentally induced strong aversion for a certain diet in rats was reduced after rats had direct contact with two conspecifics for 30 minutes each. This contact could be considered as a more intense form of social information than in our study. Thus, it is important for further studies on conformity in dogs (and other nonhuman animals) to systematically vary the intensity or value of personal and social information.

Another aspect of personal information is the information individuals receive parallel to the presented social information. In the studies of Haun et al. [11,12], observers were rewarded for stay and switch responses, whereas observers in our study were only rewarded for stay but not for switch responses. As we explained in the method section, we decided to do so because if we had rewarded both types of response, switch responses could have also been attributed to individual learning experience in past trials. In other words, if dogs showed switch responses after being rewarded for a switch response in the trial before, it would be unclear whether this switch response was due to conformity or due to the individual learning experience in the trial before. It could be argued that this logic also applies to rewarding stay responses. If dogs showed stay responses after being rewarded for a stay response in the trial before, it would be unclear whether this stay response was due to the learned preference or due to the individual learning experience in the trial before. However, not rewarding stay responses would mean removing the reward that dogs would probably anticipate because of the training. Consequently, there would be a negative punishment after a dog showed a stay response. Since one can assume that the prediction error (i.e., the violation of expectancy) for a dog is stronger in an event of negative punishment than in an event of (already anticipated) positive reinforcement, the former should have a stronger impact on the dogs’ subsequent behavior than the latter. Hence, we think that keeping the reward structure stable across training and experimental phases is preferable, because it leads to a conservative test of conformity. Nevertheless, results of recent experiments ([11,12], including ours) in which observers were rewarded in a trial wise manner should be considered with caution (irrespective of the exact reward structure), because the observed conformity was probably tainted by individual reinforcement history. A way to deal with this issue is to analyze only the responses in the first trial, as we did. Similar to our analyses over all trials, this critical analysis did not reveal evidence for conformity in dogs. Future studies should consider this issue and aim to methodologically or statistically control for the individual learning experience.

### Conformity and social information

The strength and content of the social information (i.e., the majority response) can be seen as an additional important factor contributing to the occurrence of conformity.

For example, observers in the studies of Haun et al. [11,12] could see that demonstrators were rewarded for their behavior, whereas observers in our study were prevented from seeing this. Similarly, in one condition of the study by Pike and Laland, sticklebacks were confronted with demonstrators that only shoaled but did not feed at an alternative foraging site ([15], but see [6] for a discussion of whether this was successful). In our view, presenting a reward in (spatial or temporal) contingency to an alternative response can be seen as personal information that is presented alongside the social information. This personal information could then influence the observers’ response independently of the social information. Thus, if an observer had seen the majority response alongside a reward, we cannot conclude whether a subsequent switch response was due to the fact that the observer saw the reward, due to the fact that his conspecifics showed that response, or due to both. This issue is especially relevant when the switch response is already part of the observer’s repertoire and structurally similar to the stay response, since then it is more likely that the observers simply showed a response in the direction of the reward, which is incidentally also the response of their conspecifics. Hence, preventing our observers from seeing the reward for the demonstrators enabled us to test whether dogs switched their responses due to the majority response per se. A different–less conservative–approach would have been to reward all possible responses equally and let the observers see that the demonstrators were rewarded. In this cases the effect of reward would have been held constant across response options and trials (cf. [12]. In order to investigate whether reward and social information have additive and/or interactive effects on conformity, future studies should manipulate their presence orthogonally.

In contrast to the study by Haun et al [11], but in line with the studies by Galef and Whisken [14] and by Pike et al. [15], in our study demonstrators were not present at the time when observers showed their response. Since the former study did not find evidence for conformity, whereas the latter studies did, and because humans show conformity even when they respond anonymously [3,35], there is no evidence suggesting that the presence of the majority is generally a necessary condition for conformity. Similarly, in contrast to the studies by Haun et al., observers and demonstrators were not part of the same group, which could have prevented conformity. However, since Galef and Whiskin [14], Aplin et al. and several studies on humans [3] showed that conformity can occur even when the majority is unacquainted, there is no conclusive evidence that group membership is generally a necessary condition for conformity. Testing whether the presence of and shared group membership with the majority is a necessary precondition for conformity in dogs is an interesting avenue for future research.

### Implications and outlook

All aspects of our study discussed so far suggest that the strength of the personal information was probably quite high, whereas the strength of the social information was probably quite low. In the words of van Leeuwen and Haun [9], this implies that the *distance* between personal and social information was probably quite large. Consequently, we have conducted a rather conservative test of conformity in dogs. Our results suggest that dogs do not show conformity under such conditions.

In order to test whether dogs show conformity when additional boundary conditions are met, future studies could systematically investigate the factors (and their interactions) we discussed above (i.e., strength of personal and social information, reward structure, strength of prior behavioral preference, observability of conspecifics’ response outcomes, psychological distance to majority). More generally, it is worth exploring in which situations conformity in dogs would be adaptive and whether such a situation could have occurred during dogs' evolution (i.e., in their domestication history). It is possible that conformity in dogs could have evolved when it served as a signal of group membership which facilitated social interaction and social survival [6]. Thus, when dogs were mostly held in groups and competed for resources provided by humans, it is possible that dogs evolved conformity to regulate social interaction and social order. Further research could investigate whether dogs conform to their conspecifics when this is socially adaptive. For example, it could be tested whether dogs conform to the majority of a group in order to strengthen their relation to this group (e.g., when a dog “immigrates” into a new group, or when the members of this group are present, cf. [21]).

Furthermore, it remains to be investigated whether dogs show conformist transmission. Cultural evolutionary models suggest that conformist transmission would be adaptive when the given environment underlies temporal and spatial changes leading to behavioral uncertainty [7]. Living with humans constitutes a rather stable environment, because dogs were provided with food and shelter once they had learned (or evolved) capacities that humans needed or liked. Thus, it is possible that dogs did not evolve (or lose) the tendency to copy the majority during their domestication. To test this hypothesis, future research could investigate whether naïve dogs (i.e., dogs without any personal knowledge and thus under high behavioral uncertainty) copy the majority (i.e., show conformist transmission, [2]).
